# Absorptive Roots Prioritize Chemical over Morphological Investment Under Litter Addition in a Qinling Pine–Oak Mixed Forest

**DOI:** 10.3390/plants14243768

**Published:** 2025-12-10

**Authors:** Xuehong Ma, Chengling Gong, Shuiqiang Yu, Jianhui Xue, Qian Wang, Jian Zhou, Weifeng Wang

**Affiliations:** 1 Co-Innovation Center for Sustainable Forestry in Southern China, College of Life Science, Nanjing Forestry University, Nanjing 210037, China; xuehongma@njfu.edu.cn; 2 College of Ecology and Environment, Nanjing Forestry University, Nanjing 210037, Chinayusq@njfu.edu.cn (S.Y.);; 3 Institute of Botany Jiangsu Province and Chinese Academy of Sciences, Nanjing 210014, China; jhxue@njfu.edu.cn

**Keywords:** chemical trait, morphology trait, nutrient foraging, soil variables, Qinling

## Abstract

The root economics spectrum predicts coordinated trait shifts to a heterogeneous soil environment, yet how roots strategically respond to litter-driven nutrient patches is not fully understood. We conducted a litter addition experiment (CK: 0, Low: 30, Medium: 60, High: 120 g) in a Qinling mixed forest, quantifying root responses of *Pinus tabuliformis* (Pt) and *Quercus aliena* var. *acuteserrata* (Qa). Soil inorganic nitrogen (NH_4_^+^-N and NO_3_^−^-N) increased significantly only under high litter inputs (by 138% and 130%, respectively; *p* < 0.001), indicating a threshold effect. Root carbon and nitrogen concentration generally increased under the Medium and High litter addition treatments compared to the Low treatments (*p* < 0.05), while morphological traits remained conservative (*p* > 0.05). Species identity showed no significant effect in the multivariate root trait syndrome (redundance analysis, *p* = 0.716), though species-specific responses were observed in the root carbon concentration (Pt: *p* < 0.05; Qa: n.s.). These results demonstrate a hierarchical foraging strategy where physiological plasticity dominates over morphological change, challenging the root economics spectrum and providing a multidimensional framework for predicting root function in heterogeneous environments.

## 1. Introduction

Mixed forests, characterized by the coexistence of functionally distinct tree species (e.g., conifers and broadleaves), are dominant in temperate mountain ecosystems such as China’s Qinling Mountains, serving as critical hubs for biodiversity conservation and ecosystem functionality (e.g., carbon/C sequestration) [1,2,3,4]. The availability of essential nutrients, particularly nitrogen (N) and phosphorus (P), is frequently limited in these mountain mixed forest regions due to high soil heterogeneity and complex interactions between climate, topography, and biological processes [5]. Within this challenging context, plant litter decomposition serves as a fundamental process in forest nutrient cycling, especially nitrogen cycling [6,7]. Soil heterogeneity is amplified by patchy litter distribution (nutrient-rich patches and -poor patches) [8]. Changes in root production and turnover in response to anthropogenic environmental changes represent a critical link between plant responses and longer-term alterations in soil biogeochemical dynamics [9]. Understanding how root functional traits—particularly the decoupling of chemical and morphological dimensions—respond to nutrient enrichment is essential to predict the belowground consequences of global change.

Litter quality and quantity are fundamental drivers of ecosystem nutrient cycling. The quantity of litterfall directly determines the soil fauna diversity and the nutrients returned to the soil via litter decomposition [10,11,12]. Current understanding of litter–soil nitrogen dynamics remains contradictory. While some studies report increased nitrogen availability [13,14], others demonstrate that fresh litter stimulates microbial immobilization, temporarily reducing plant-accessible nitrogen [10,15]. This conflict suggests a non-linear relationship governed by a critical litter input threshold. This interplay creates a heterogeneous soil environment characterized by spatially and temporally variable nutrient patches, posing a dynamic challenge for plants to optimize their nutrient acquisition through root trait adjustments.

In response to such soil resource heterogeneity, plants are not passive recipients but active foragers [16]. They dynamically adjust their belowground investment through changes in root morphological traits, a phenomenon known as root foraging plasticity [16,17]. Key traits such as specific root length (SRL), root tissue density (RTD), and average root diameter (ARD) collectively define a plant’s foraging strategy within the conceptual framework of the root economics spectrum [18,19]. This spectrum encapsulates a trade-off between resource acquisition and conservation, ranging from an acquisitive strategy (characterized by high SRL, low RTD, and thin roots for rapid resource uptake) [20,21,22] to a conservative strategy (featuring low SRL, high RTD, and thick roots for long lifespan and resource preservation) [21,23,24,25]. Nonetheless, growing evidence suggests that root trait coordination may be multidimensional, forming a ‘root economics space’ that extends beyond the classical fast–slow trade-off [19]. Assessing root responses within this multidimensional framework may provide a more mechanistic understanding of foraging strategies. Therefore, understanding the response and shift in species foraging strategies on litter-induced nutrient-rich patches along this spectrum trait is crucial for deciphering their belowground resource-use strategies.

While it is well-established that plants can modify their root traits in response to nutrient-rich patches [16,26,27,28,29]. Litter decomposition, as a core driver of nutrient cycling [30,31], creating localized “nutrient-rich patches” through asynchronous release of essential elements (e.g., N and P) from heterogeneous litter distribution. This process reshapes the spatial and temporal distribution of soil resources, providing critical shifts in plant root foraging strategies. However, it remains unclear whether roots respond to such litter-mediated nutrient patches in a coordinated manner, as predicted by the root economics spectrum, or through a more complex, hierarchical strategy where morphological and chemical traits respond independently. This knowledge gap hinders our understanding of how plants dynamically adapt to litter-mediated resource variability in mountain forests, particularly under changing environmental conditions that alter litter dynamics.

To address this knowledge gap, we conducted a litter addition experiment in a temperate pine–oak mixed forest on the Qinling Mountains, China. The objective of this study was to mechanistically unravel how litter-induced nutrient imbalances (nutrient-rich patches) influence the root foraging traits of two co-dominant tree species, *Pinus tabuliformis* (Pt) and *Quercus aliena* var. *acuteserrata* (*Qa*). We hypothesized that (H1) litter addition would not induce a linear increase but a threshold response in soil nitrogen availability and (H2) root foraging strategies would exhibit an asymmetric response, with chemical traits demonstrating greater plasticity than morphological traits.

## 2. Results

### 2.1. Soil Nutrient Pool Under Litter Addition

**Figure 1:**
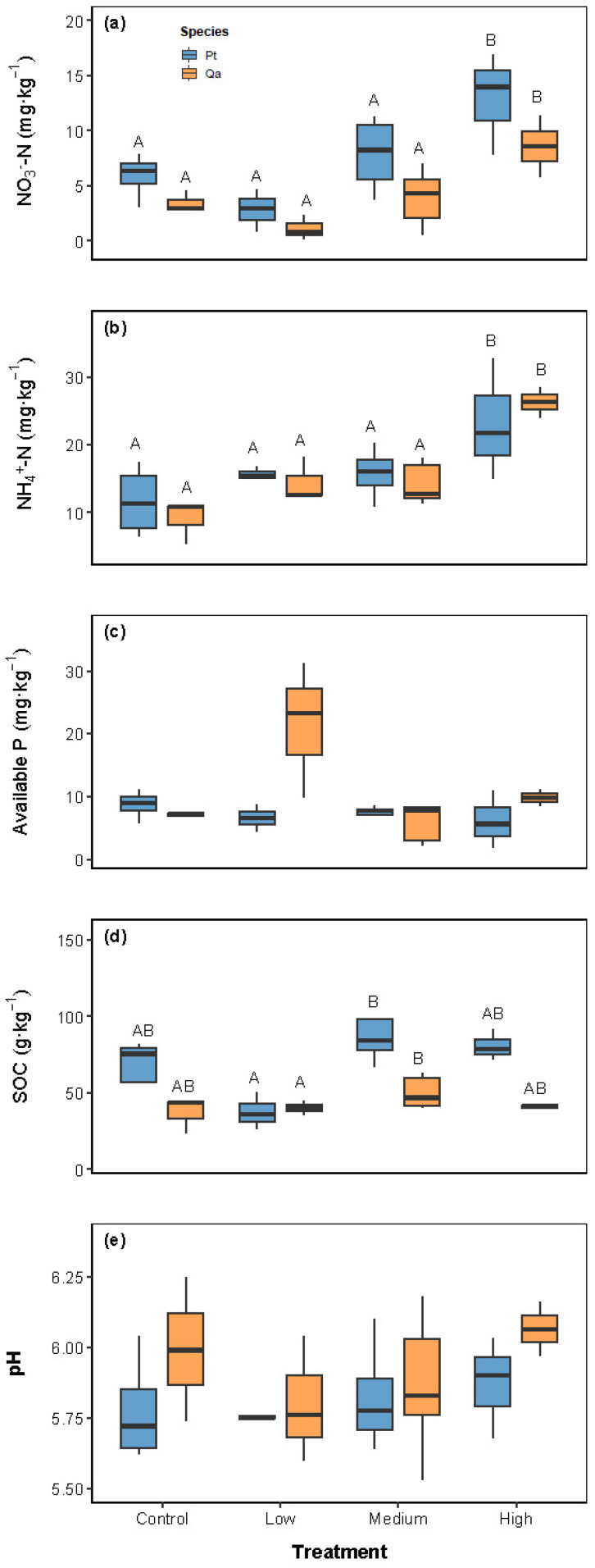
Comparisons of soil properties, including NO_3_^−^-N (**a**), NH_4_^+^-N (**b**), available P (**c**), soil organic carbon (**d**), and pH (**e**), among litter addition treatments (CK 0 g; Low: 30 g; Medium: 60 g, High: 120 g). Blue bars represent *Pinus tabuliformis* (Pt) and yellow bars represent *Quercus aliena* var. *acuteserrata* (Qa). Different uppercase letters indicate significant differences among treatments (*p* < 0.05). Panels without letters show no significant effects (*p* > 0.05).

Soil nutrient pools varied in response to experimental litter addition, with distinct patterns observed across different nutrient pools (Table 1 and Figure 1). Litter addition exerted a significant overall effect on NH_4_^+^-N and NO_3_^−^-N levels, with high (120 g) litter inputs leading to notable increases compared to the CK (Figure 1a,b).

In contrast, available P (AP) and soil pH showed no significant responses to litter addition (all *p* > 0.05; Table 1 and Figure 1c,e). Notably, species identity (Pt vs. Qa) had no significant influence on any measured soil variables across treatments (all *p* > 0.05).

### 2.2. Response of Root Trait to Litter Addition

**Figure 2:**
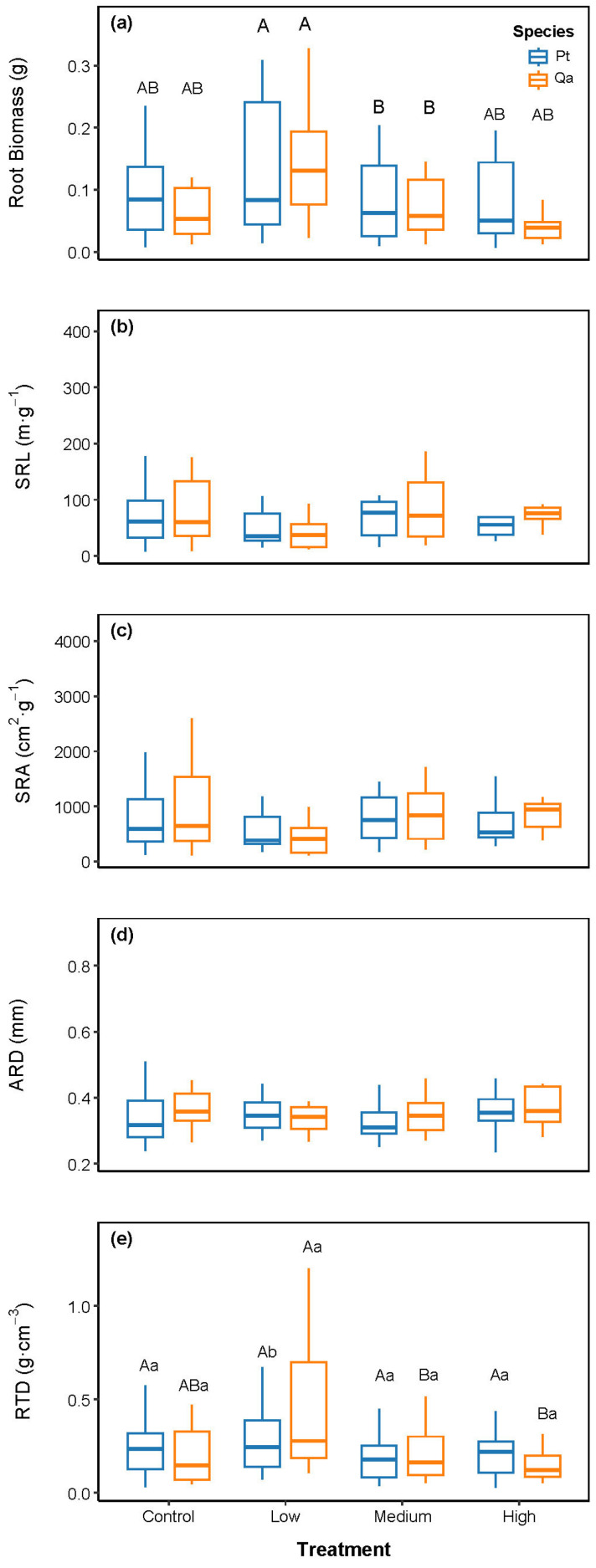
Comparisons of absorptive root biomass (**a**) and morphological traits, including SRL (**b**), SRA (**c**), ARD (**d**), RTD (**e**), among litter addition treatments (CK 0 g; Low: 30 g; Medium: 60 g, High: 120 g). Blue bars represent *Pinus tabuliformis* (Pt) and yellow bars represent *Quercus aliena* var. *acuteserrata* (Qa). Different uppercase letters indicate significant differences among treatments (*p* < 0.05). Lowercase letters indicate significant differences between species within treatments (*p* < 0.05). Panels without letters show no significant effects (*p* > 0.05).

Linear mixed models revealed that litter addition significantly altered root biomass and key chemical traits, shifting them toward a more acquisitive foraging strategy. Specifically, root biomass increased significantly under the Low litter addition treatment compared to CK (Figure 2a; *p* < 0.05), whereas no significant change was observed in Medium and High treatments relative to CK (*p* > 0.05). RTD generally remained unchanged across treatments, except for a significant increase in Qa under the Low treatment (Figure 2e, *p* < 0.05). In contrast, SRL, SRA, and ARD were not significantly influenced by litter addition (Figure 2b–d).

**Figure 3:**
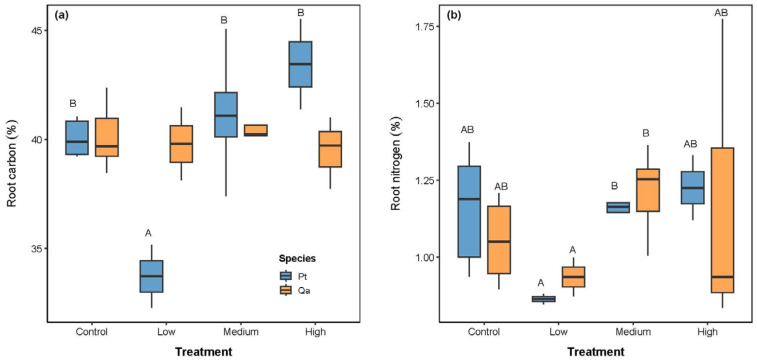
Comparisons of absorptive root chemical traits, including carbon (**a**) and nitrogen (**b**), among litter addition treatments (CK 0 g; Low: 30 g; Medium: 60 g, High: 120 g). Blue bars represent *Pinus tabuliformis* (Pt) and yellow bars represent *Quercus aliena* var. *acuteserrata* (Qa). Different uppercase letters indicate significant differences among treatments (*p* < 0.05).

Root C concentration increased significantly with litter addition for Pt (*p* < 0.05, Table 2 and Figure 3a), while it had no significant change in Qa. Root N concentration responded positively to litter addition. It increased significantly under the Medium and High treatments (both 1.20%) compared to the Low treatment (Low: 0.89%, *p* < 0.05, Figure 3b). However, no significant changes were detected between the CK and the Medium and High treatments (*p* > 0.05).

### 2.3. Multivariate Response of Root Trait Syndrome

**Figure 4:**
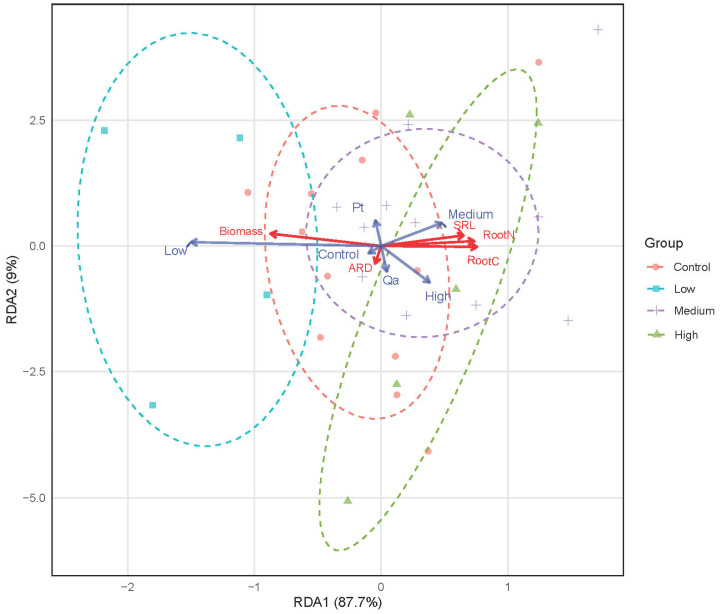
Divergence in absorptive root trait syndromes in response to a gradient of litter addition (Control 0 g; Low: 30 g; Medium: 60 g, and High: 120 g). Pt and Qa represent *Pinus tabuliformis* and *Quercus aliena* var. *acuteserrata,* respectively. ARD: average root diameter. SRL: specific root length. Root trait vectors are shown in red. Centroids for constraint variables (litter addition treatments and tree species) are shown in blue.

The redundancy analysis (RDA) revealed a powerful effect of litter addition on the multivariate root trait syndrome (F = 2.238, *p* = 0.019, Figure 4). The first RDA axis (RDA1) explained 87.7% of the constrained variance and formed a clear gradient from Control to High litter addition treatments. The Medium and High litter addition treatments clustered on the right, strongly associated with the acquisitive strategy traits of higher SRL and root nitrogen concentration. The trait vectors for SRL and root N pointed strongly in the same direction along the treatment gradient. However, the effect of tree species was not significant in the multivariate space (F = 0.505, *p* = 0.716).

The univariate and multivariate results are fully consistent. The significant increases in root nitrogen from Low treatments to Medium and High treatments, coupled with the decrease in root biomass identified by the LMMs, are precisely the changes that drive the major ecological strategy gradient captured by the RDA. The lack of a significant species effect in the response highlights a convergent physiological foraging strategy when nutrients are abundant.

## 3. Discussion

### 3.1. Litter Addition-Induced Shifts in Soil Nutrient Availability

Our results demonstrate that litter addition did not induce a linear increase in soil nitrogen availability but instead exhibited a clear threshold effect, supporting our first hypothesis. The observed initial decline or non-significant change in soil inorganic nitrogen under Low and Medium litter additions in our field study (Figure 1a,b) can be mechanistically explained by the phenomenon of microbial nitrogen immobilization. This is strongly supported by a controlled incubation experiment with litter from various Chinese fir (*Cunninghamia lanceolata)* clones, which demonstrated that net rates of N mineralization and nitrification decreased linearly with increasing litter addition rates [15]. Their findings confirm that the input of fresh, carbon-rich litter stimulates microbial activity, which preferentially immobilizes available mineral nitrogen, thereby creating a transient but significant nitrogen sink that reduces immediate plant-available N pools. A study conducted by Lu et al. [10] documented that in the high-diversity subtropical montane forest, litter manipulation only altered invertebrate communities with no soil nutrient responses, whereas in the low-diversity tropical lowland forest, both soil nutrients and invertebrates responded to litter quantity. This potentially occurs via more complex plant–microbe–invertebrate interactions that stabilize nutrient pools in the high-diversity subtropical montane forest. This pattern strongly suggests the existence of a critical litter input quantity that must be exceeded to shift the soil microbial process from net immobilization to net mineralization.

However, litter addition stimulates both net nitrite and net ammonification rate in a Masson pine (*Pinus massoniana*) pure forest and in a Masson pine and Camphor (*Cinnamomum* sp.) tree mixed forest of subtropic regions [14]. A study on litter manipulation effects on soil N dynamics in a *Larix gmelinii* boreal forest found that, relative to the control, litter addition increased average soil inorganic N content, microbial biomass N (MBN), and net N mineralization rate in the 0–20 cm layer by 40.58%, 54.16%, and 128.57%, respectively [13]. High litter quantity may favor macro- over meso-detritivores in agricultural soils, which would influence food webs [11] and, thereby, litter decomposition and soil nutrient dynamics. This contrast strengthens the complexity of interactions between litter decomposition and soil nutrient dynamics in such mountain ecosystems. These findings underscore the complex interplay between litterfall, soil microorganisms, and nutrient cycling, which emphasizes the need to consider these interactions in ecosystem management, potentially via more complex plant–microbe–invertebrate interactions that stabilize nutrient pools.

### 3.2. Asymmetric Trait Response in Absorptive Roots to Soil Nutrients

Plants actively optimize nutrient acquisition in heterogenous soil environments through localized root proliferation in nutrient-rich patches, such as those derived from litter decomposition, along with morphological plasticity like SRL [32,33,34]. Against this theoretical background, our results reveal a fundamental dichotomy in how absorptive roots respond to litter-mediated changes in soil nutrient availability. Morphological traits exhibit remarkable conservatism, while chemical traits demonstrate significant plasticity. This asymmetric response pattern challenges the expectation of coordinated shifts along a root economics spectrum and highlights a hierarchy of strategies in root foraging, partially supporting our second hypothesis. This morphological inertia found in our study is consistent with findings from desert shrub communities, where root traits failed to co-vary with soil nutrient availability despite clear leaf economic patterns [35], and fine root morphology remained largely unchanged under one-year nitrogen addition in a subtropical *Mytilaria laosensis* plantation [36]. However, studies showed that Chinese fir increased root length but decreased SRL in nutrient-rich patches, whereas bamboo exhibits the opposite response, increasing SRL [37], and the species-specific responses of root traits to soil nutrients were common in karst ecosystems [38]. Therefore, this decoupled response highlights a foraging strategy within absorptive roots: adjustments to nutrient acquisition were driven primarily through changes in physiological state rather than through modifications to morphological form.

The discrepancy between our non-significant linear models for individual traits (e.g., Figure S2), and the strong and coordinated shift in the multivariate trait syndrome is particularly instructive (Figure 4). This pattern challenges simplistic root economics spectrum models and aligns with the emerging paradigm of complex multidimensional root foraging strategies [26]. The morphological conservatism likely represents a strategic choice under carbon constraints, mirroring stand-development trade-offs where declining SRL is compensated by increased root N concentration [39]. The fact that this integrated syndrome was only detectable through multivariate analysis highlights how coordinated trait responses to environmental heterogeneity can be obscured in univariate approaches [40].

### 3.3. Ecological Implications

Our findings, revealing a fundamental decoupling between morphological conservatism and chemical plasticity in absorptive roots, challenge the unidimensional “fast–slow” paradigm of the root economics spectrum [18]. The classic root economics spectrum posits a suite of coordinated traits along a single acquisition–conservation axis, a framework that has been broadly extended to envision a whole-plant economics spectrum [18]. However, our results align with a growing body of evidence suggesting that root trait coordination is more complex and multidimensional. The observed morphological inertia, despite shifts in soil nutrients, resonates with studies in desert communities where root traits failed to co-vary with soil fertility, disrupting the expected aboveground–belowground trait synergy [35]. This dissonance can be partly explained by non-linear allometric relationships between root tissues, which complicate simple trade-offs and can lead to conflicting interpretations of the root economics spectrum depending on sampling bias [41]. Therefore, our study strengthens the emerging paradigm of a multidimensional root economics space, where traits governing resource conservation (e.g., morphology) can vary independently from those related to physiological activity and fungal collaboration [19]. The fact that this integrated strategy was only detectable through multivariate analysis underscores that unidimensional models often obscure the hierarchical and context-dependent nature of root foraging strategies in heterogeneous environments.

### 3.4. Limitations and Future Perspectives

This study provides valuable insights into root foraging strategies in response to litter heterogeneity, yet several limitations should be acknowledged. The primary limitation is the unbalanced litter addition experimental design and the relatively low number of replicates for chemical traits measurements, while LMM + ANOVA III and data are sufficient for detecting strong patterns using multivariate techniques like RDA. Future studies would benefit from a more robust sample size. Secondly, the one-growth-season experiment captures only short-term responses; long-term (3–5 years) litter accumulation may alter soil C/N ratios and microbial community composition. Finally, the scope of our study was limited to root traits, and the contribution of mycorrhizal fungi remains an unmeasured component of the observed foraging strategies.

Therefore, future research should address the temporal, biological, and climatic complexities of root foraging. First, long-term experiments are needed to track how nutrient pulses sequentially drive root trait plasticity and soil nitrogen transformations over multiple years, as the legacy effects of such perturbations can fundamentally alter ecosystem recovery trajectories [42]. Second, the critical role of soil microbiota must be incorporated. We recommend that future work dissects how the ‘extended root phenotype’ is governed by mycorrhizal type [43]. This will functionally link plant resource acquisition syndromes to the activity and composition of the rhizosphere microbiome [44,45]. Finally, linking belowground foraging efficiency with remotely sensed canopy spectral indices (e.g., NDVI, chlorophyll content) could provide a powerful, non-invasive proxy for monitoring root function and forest health across landscapes. Experimental simulations of interactive global change factors, such as drought under warming scenarios, will test the resilience of the observed root foraging hierarchies and determine how climate extremes might reshape belowground carbon and nutrient cycling [42].

## 4. Materials and Methods

### 4.1. Study Area

The study was carried out at Xinkuang Forest Farm (33°20′–33°26′ N, 108°32′–108°34′ E), which is administrated by Ningdong Forestry Bureau in Shaanxi Province, China. Geographically, it is situated at the southern Qinling Mountains, with elevation ranging from 1095 to 2591 m, belonging to the management region of subtropical evergreen broad-leaved forests and coniferous–broad-leaved mixed forests in southern China. The climate is a north subtropical semi-humid monsoon climate, with four distinct seasons. The average annual temperature is 8.5 °C, and annual precipitation reaches 908 mm, with over 70% of rainfall concentrated in the growing season (May–September). Soils are primarily mountain yellow-brown earths, featuring a clay-like texture and a typical depth of 30–60 cm; the topsoil (0–20 cm) has a slightly acidic to neutral pH (5.5–6.8).

The studied stands are secondary mixed forests that naturally regenerated following logging activities in the 1970s. The dominant tree species include Pt, Qa, and *Pinus armandii*, which form the main canopy layer (15–20 m in height). Associated tree species in the subcanopy include *Toxicodendron vernicifluum*, *Ligustrum quihoui*, and *Acer davidii*. The understory vegetation is dominated by shrubs such as *Euonymus alatus* and *Litsea pungens*, along with herbs including *Rubus* spp., *Carex canteolata*, *Agrimonia pilosa*, and *Rubia cordifolia*. We selected 6 healthy Pt and 6 healthy Qa individuals with no pests or diseases. The selected plants had DBH (diameter at breast height) of Pt ranging 16.7–37.8 cm, and Qa ranging 14.2–26.2 cm, to represent individual growth differences on the experimental results.

### 4.2. Litter Addition Treatments and Experimental Design

This study employed a factorial design with two factors: tree species and litter treatment. At the beginning of the growing season (May 2024), initial soil cores (0–15 cm depth) were collected using a root auger (7 cm in diameter) from the crown projection area of 12 sample trees (6 per species) at 1 m from the trunk. Four cores were taken per tree, one in each cardinal direction (south, west, north, and east), at locations free of understory vegetation (i.e., at least 1 m away from shrubs, seedlings, and large herbaceous plants).

The soil cores were sieved through a 5 mm mesh to remove roots, gravel, and other impurities. The resulting root-free soil was reserved for filling the growth bags, while the sieved roots were collected and transported to the laboratory to establish a reference for initial root background data.

Litter addition gradients were designed based on the local annual litterfall (620 g m^−2^ yr^−1^) and the existing litter layer mass (1574 g m^−2^). The equivalent litter dry mass for a single growth bag (basal area = 0.003848 m^2^) was calculated to be 14.5 g. To determine the corresponding fresh litter mass, the natural fresh litter mass per bag was 14.5 g × (1 + 1.38) ≈ 34.5 g. Four treatments were established to simulate a range from natural input to accumulated conditions: a control (CK) with root-free soil only; a 30 g treatment, approximating the natural litter mass; and elevated 60 g and 120 g treatments. These higher levels were designed to test for potential shifts in root foraging strategies under litter inputs that exceed the ecosystem’s typical decomposition capacity.

The CK and 60 g treatments were each applied to 6 trees (3 per species), while the 30 g and 120 g treatments were each applied to 3 trees. This allocation resulted in a total of 144 growth bags: 48 for CK, 24 for 30 g, 48 for 60 g, and 24 for 120 g. Around each tree, four bags (one per treatment) were deployed in the four cardinal directions. All bags were vertically buried with their openings flush with the soil surface in the 0–15 cm layer, and their positions were marked.

### 4.3. Sampling and Measurements

In November 2024 (6 months after the placement of growth bags), all 144 growth bags were completely retrieved. Roots and soil were separated from the growth bags: root samples were picked out with tweezers, and live roots were distinguished from dead roots based on color (live roots are white or light yellow-brown, while dead roots are dark brown or black) and elasticity (live roots are tough, while dead roots are brittle). Live roots were retained for subsequent determination. Soil samples were air-dried naturally, sieved through a 2 mm mesh, and sealed for soil nutrient analysis.

#### 4.3.1. Fine Root Indicator Determination

Root samples were rinsed with distilled water until few soil remained. Roots were classified using the root order method, where root orders 1–2 were defined as absorptive roots and orders 3–5 as transport roots [46]. Only absorptive roots (orders 1–2) were retained for subsequent trait measurements. The classified roots were scanned using a digital scanner (10000XL 1.0, Expression, Washington, DC, USA), and the scanned images were analyzed using Win RHIZO (Pro 2005b) root image analysis software (Regent Instruments Inc, Québec, QC, Canada) to determine morphological indicators, including average diameter, total length, total surface area, total volume, and fine root number. After determination, the roots were oven-dried at 65 °C for 72 h to constant weight (WF-11E, Wiggens, Straubenhardt, Germany), and fine root biomass was weighed on an electronic balance (BSA323S, Sartorius, Göttingen, Germany). C and N contents in fine roots, that we combined from four direction samples together, were determined using an elemental analyzer (2400 Series II, PerkinElmer, Waltham, MA, USA). Root biomass and morphological traits were recorded as ‘0’ when no live roots were observed in the growth bag (i.e., no root colonization). We observed that 14.9% of bags had no root colonization. These zero values were retained in the dataset to reflect root foraging absence, rather than excluded as missing data.

#### 4.3.2. Soil Indicator Determination

Soil organic C (SOC) was determined using the potassium dichromate oxidation–external heating method. Soil ammonium N (NH_4_^+^-N) was extracted with 2 mol/L KCl (soil-to-water ratio 1:5), and the extract was determined using the indophenol blue colorimetric method. Soil nitrate N (NO_3_^−^-N) was extracted with 2 mol/L KCl (soil-to-water ratio 1:5), and the extract was determined using the ultraviolet spectrophotometry (wavelengths of 220 nm and 275 nm). AP was extracted with 0.5 mol/L NaHCO_3_ (soil-to-water ratio 1:20), and the extract was determined using the molybdenum–antimony anti-colorimetric method.

### 4.4. Statistical Analyses

All statistical analyses were performed in R 4.5.1 (R Core Team, 2023). Normality was tested via Shapiro–Wilk tests, and homogeneity of variance via Levene’s tests. Skewed data can be transformed (log_10_) to meet parametric assumptions. To address the unbalanced experiment design (unequal sample sizes across treatments), we employed linear mixed effects models (LMMs) with sample trees as a random effect for soil variables (NH_4_^+^-N, NO_3_^−^-N, AP, SOC, and pH) and root traits such as biomass, morphology (SRL, SRA, RTD, and ARD), and chemistry (C and N) via the “lme4” packages [47]. Type III ANOVA was used to access the significance of fixed effects (litter addition, tree species, and their interactions), followed by Tukey’s HSD test.

To assess the integrated responses of the multivariate root trait syndrome to litter additions, we performed an RDA. To address the strong collinearity among root variables before conducting RDA, the most common and effective Pearson method was used to assess pairwise correlations and remove redundant variables based on correlation strength (Figure S1). A general threshold is to consider variables as “strongly collinear” if their absolute correlation coefficient (|r|) exceeds 0.7. Finally, the five root traits including root biomass, root C concentration, root N concentration, SRL, and ARD were used as the response matrix. The explanatory variables were the litter addition treatment (treatment: Control, Low, Medium, High) and tree species (Species: Pt, Qa), both included as fixed factors. The significance of the global model and of each constrained term (treatment and species) was tested using permutation tests (999 permutations) under a reduced model. The analysis was performed using the “vegan” package [48].

## 5. Conclusions

This study demonstrates that trees employ a hierarchical foraging strategy where absorptive roots respond to nutrient heterogeneity primarily through physiological plasticity rather than morphological restructuring, challenging the traditional root economics spectrum. This mechanistic understanding provides a functional basis for selecting resilient tree species in forest restoration and managing litter inputs to directly steer soil nutrient cycling. To advance these findings, future research should integrate long-term experiments and plant–fungal networks with remote sensing technologies, enabling us to scale these individual-level foraging strategies to predict ecosystem-level resilience under climate change.

## Figures and Tables

**Table 1:** Linear mixed effects model (LMM) Type III ANOVA results of soil variables affected by litter addition and tree species.

Soil Variable	Sample Size (n)	Litter Addition	Tree Species	Litter × Species Interaction
NO_3_^−^-N	27	**F_3_** _, 8.67_ = 17.313 ***	F_1, 9.22_ = 1.490	F_3, 9.22_ = 1.581
NH_4_^+^-N	27	**F_3_** _, 16.67_ = 9.998 ***	F_1, 10.62_ = 0.094	F_3, 16.67_ = 0.443
AP	27	F_3, 19_ = 0.555	F_1, 19_ = 0.213	F_3, 19_ = 2.448
SOC	27	**F_3_** _, 19_ = 5.212 **	F_1, 19_ = 2.701	F_3, 19_ = 1.830
pH	27	F_3, 11.73_ = 0.214	F_1, 8.57_ = 0.767	F_3, 11.73_ = 0.105

Statistical analyses were performed using linear mixed effects models (LMMs) with (1|TreeID) as random effects to account for nested data structure (data nested within tree individuals). Type III ANOVA was performed with Satterthwaite’s method for denominator degree of freedom. If random effects were close to zero, a linear model was used. Degrees of freedom (df) were presented as numerator, denominator. Significance values in bold:,** *p* < 0.01, *** *p* < 0.001.

**Table 2:** Linear mixed effects model (LMM) Type III ANOVA results of root traits affected by litter addition and tree species.

Trait Type	Trait	Sample Size (n)	Litter Addition	Tree Species	Litter × Species Interaction
Biomass	Biomass	139	**F_3__, 88.34_** **=** 4.307 **	F_1, 10.12_ = 0.047	F_3, 88.34_ = 1.479
Morphological	Average root diameter	139	F_3, 89.25_ = 1.282	F_1, 10.19_ = 0.261	F_3, 89.25_ = 0.343
traits	Root tissue density	139	F_3, 89.05_ = 2.430	F_1, 9.49_ = 0.242	**F_3__, 89.05_** **=** 3.279 *
	Specific root area	139	F_3, 131_ = 0.603	F_1, 131_ = 0.030	F_3, 131_ = 0.022
	Specific root length	139	F_3, 131_ = 0.770	F_1, 131_ = 0.008	F_3, 131_ = 0.038
Chemical traits	Root carbon	32	**F_3__, 15.65_** **=** 3.316 *	F_1, 10.96_ = 0.062	**F_3__, 15.65_** **=** 4.447 *
	Root nitrogen	32	**F_3__, 17.11_** = 4.148 *	F_1, 12.03_ = 0.021	F_3, 17.11_ = 0.0474

Degrees of freedom (df) are presented as numerator, denominator. Significance values in bold: * *p* < 0.05, ** *p* < 0.01.

## Data Availability

Data will be made available on request.

## Supplementary Materials

The following supporting information can be downloaded at: https://www.mdpi.com/article/10.3390/plants14243768/s1, Figure S1: Pearson correlation heatmap showing relationships among soil physicochemical properties, nutrient availability, and root morphological and chemical traits; Figure S2: Linear mixed model estimates of absorptive root trait responses to soil ammonium nitrogen (NH_4_^+^-N) availability.
