# Supplementary material for: Absorptive Roots Prioritize Chemical over Morphological Investment Under Litter Addition in a Qinling Pine–Oak Mixed Forest
Source: Plants (Basel). 2025 Dec 10;14(24):3768. doi: 10.3390/plants14243768 (PMC12736430; doi:10.3390/plants14243768)
Supplement: Supplementary file 1 [file plants-14-03768-s001.zip › plants-4003717-supplementary.pdf]

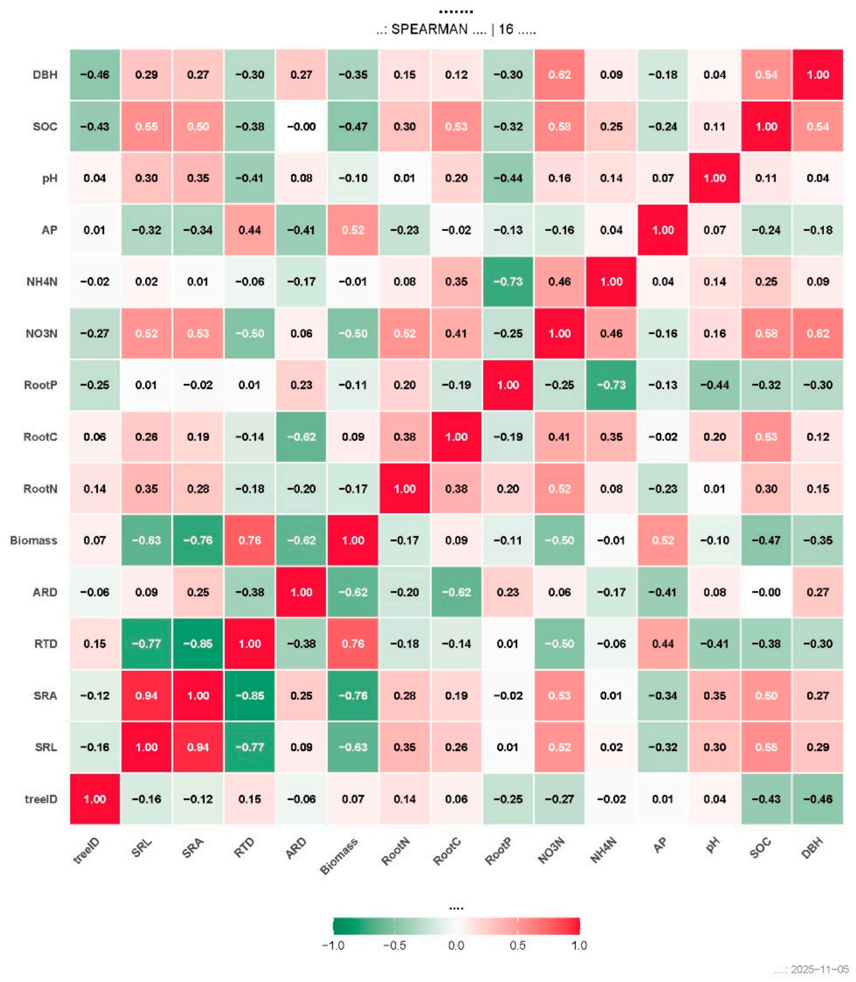

Figure S1. Pearson correlation heatmap showing relationships among soil physicochemical properties, nutrient availability, and root morphological and chemical traits. Red indicates positive correlations, green indicates negative correlations, and white indicates no significant correlation. SOC- Soil Organic Carbon (%)； AP- Available Phosphorus (mg/kg); NH4N-Ammonium Nitrogen (mg/kg); NO3N- Nitrate Nitrogen (mg/kg); ARD- Average Root Diameter (mm); SRA**-** Specific Root Area (cm^2/g); SRL**-** Specific Root Length (m/g); RTD**-** Root Tissue Density (g/cm^3); biomass**-** Root Biomass (g); RootC**-** Root Carbon Content (%)**;** RootN- Root Nitrogen Content (%).

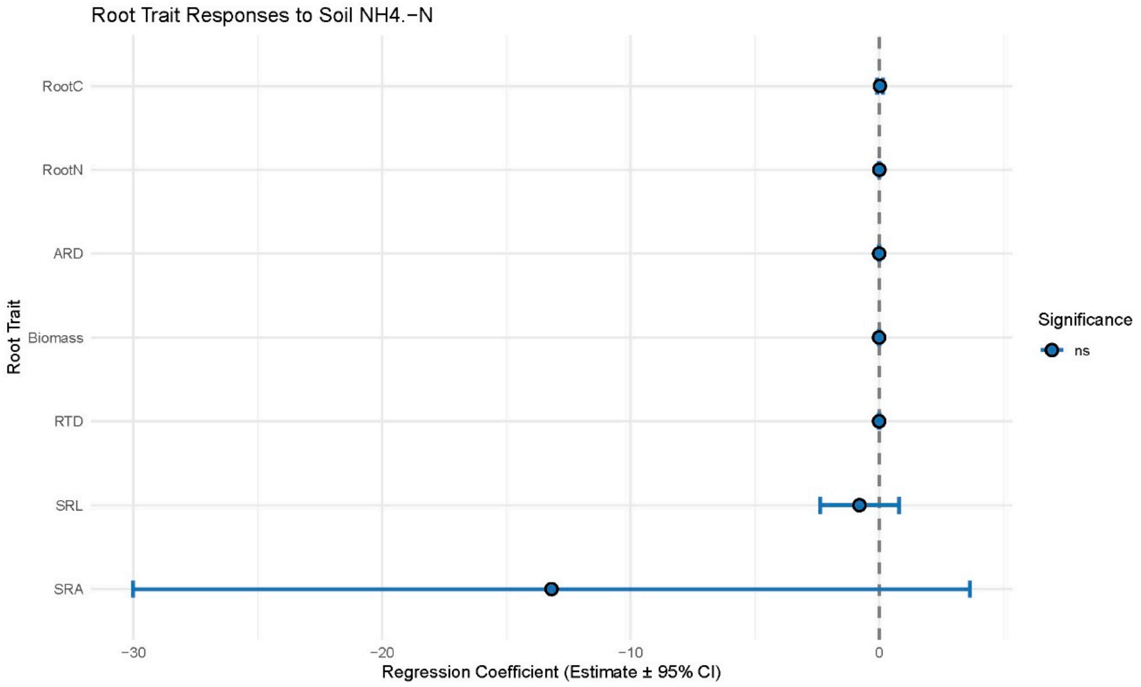

Figure S2. Linear mixed model estimates of absorptive root trait responses to soil ammonium nitrogen (NH_4^+-N) availability. RootC**-** Root Carbon Content (%)**;** RootN**-** Root Nitrogen Content (%); ARD- Average Root Diameter (mm); RTD**-** Root Tissue Density (g/cm³); SRA**-** Specific Root Area (cm²/g); SRL**-** Specific Root Length (m/g);
